# Towards the First Generation of Biomimetic Fixation for Resurfacing Arthroplasty Endoprostheses

**DOI:** 10.3390/biomimetics9020099

**Published:** 2024-02-08

**Authors:** Ryszard Uklejewski, Mariusz Winiecki, Mikołaj Dąbrowski, Piotr Rogala

**Affiliations:** 1 Department of Constructional Materials and Biomaterials, Faculty of Materials Engineering, Kazimierz Wielki University, Jan Karol Chodkiewicz Street 30, 85-064 Bydgoszcz, Poland; 2 Adult Spine Orthopaedics Department, Wiktor Dega Orthopaedic and Rehabilitation Clinical Hospital, Poznan University of Medical Sciences, 28 Czerwca 1956 Street 135/147, 61-545 Poznan, Poland; mdabrowski@ump.edu.pl; 3 Institute of Health Sciences, Hipolit Cegielski State College of Higher Education, Card. Stefan Wyszyński Street 38, 62-200 Gniezno, Poland; gabinet.rogala@gmail.com

**Keywords:** resurfacing arthroplasty, resurfacing endoprostheses, biomimetic multi-spiked connecting scaffold (MSC-Scaffold), biomimetic fixation

## Abstract

This paper presents advances in designs of resurfacing arthroplasty endoprostheses that occurred through their historical generations. The critical characteristics of contemporary generation hip resurfacing arthroplasty endoprostheses are given and the failures resulting from the specific generation cemented and short stem fixation of the femoral component are reviewed. On the background of these failures, the critical need arises for an alternative approach to the fixation of components of resurfacing arthroplasty leading towards the first generation of biomimetic fixation for resurfacing arthroplasty endoprostheses. The state of the art of the completed bioengineering research on the first biomimetic fixation for resurfacing arthroplasty endoprostheses is presented. This new design type of completely cementless and stemless resurfacing arthroplasty endoprostheses of the hip joint (and other joints), where endoprosthesis components are embedded in the surrounding bone via the prototype biomimetic multi-spiked connecting scaffold (MSC-Scaffold), initiates the first at all generations of biomimetic endoprostheses of diarthrodial joints.

## 1. Introduction

Hip resurfacing arthroplasty (HRA) involves replacing the femoral head acetabular articular cartilage, and subchondral bone with prosthetic components designed to replace the removed articular cartilage and subchondral periarticular bone to minimize the change in overall joint kinematics [1]. The important benefit of advocating for HRA over the long-stem total hip arthroplasty (THA) is the possibility of preserving at the initial operation a bone stock. Replacing diseased tissue with near anatomic-sized femoral component retains the potential for revision since the femoral canal was not violated [2,3]. Along with the bone tissue preservation, the HRA design solutions and applied fixation techniques are assumed, contrary to the long-stem THA systems, to allow near-physiological load transfer in periarticular bone allowing the recreation of closely native hip kinematics and bone biomechanics [4]. With its excellent functional outcome [5,6,7], HRA remains a reasonable alternative to THA in the appropriate patient cohort [8], and by many surgeons, it is considered an excellent option for hip reconstruction in young patients and/or high activity level patients diagnosed with osteonecrosis of the femoral head or acetabular dysplasia [9].

The contemporary generation of HRA endoprostheses has been utilized for over 20 years, while resurfacing arthroplasty has a hundred-year-long history [10,11,12,13]. From its beginning, the concept of resurfacing arthroplasty evolved through the variety of designs of endoprosthesis components, different material choices used and through the changes in fixation methods. Through the decades, its success has varied widely and was always limited by specific technological limitations of particular eras. The registered failures of resurfacing arthroplasty endoprostheses coupled with new technological capabilities are the foundations of occurring innovations and indications for the new designs. To achieve successful long-term results, apart from design features of resurfacing arthroplasty endoprostheses and applied manufacturing processes, the key importance also are patient selection and surgeon experience [9].

This review aims to present chronologically the background of the chronological milestones through the past consecutive design generations of resurfacing arthroplasty endoprostheses and critical insight into contemporary HRA endoprostheses, the state-of-art bioengineering research on the first generation of biomimetic fixation for resurfacing arthroplasty endoprostheses.

## 2. Milestones of Early Materials and Designs of Resurfacing Arthroplasty Endoprostheses

The first design considered to be the start basis for later-known HRA was devised in 1923 by Smith-Petersen and applied to regenerate worn and damaged articular cartilage [14]. The thin, ball-shaped, hollow hemisphere manufactured of glass, which fits over the ball of the hip joint, was placed between the femoral head and the acetabulum and intended to stimulate cartilage regeneration on both sides of the moulded glass joint. The glass shell was supposed to be removed after the restoration of the cartilage. Even though the glass was biocompatible and provided a smooth surface for motion, the system failed immediately because it could not withstand physiological weight-bearing stress. Different types of glass with improved properties were used in further developed versions of this design, leading to attempts with other materials, such as Viscaloid (a derivative of celluloid) in 1925, Pyrex in 1933, and Bakelite in 1937 [15]. The performance of these solutions was far from satisfactory, causing severe inflammatory reactions due to material wear debris in the joint. The glass mould arthroplasty could not withstand weight-bearing pressure and failed shortly after surgery [14].

Around the same time, Groves introduced one of the first ivory hip arthroplasties [16]. In 1927, he replaced the femoral head in an ankylosed hip with a stemmed ivory head replacement. The design was similar to contemporary HRA endoprostheses [17].

In the 1930s, Vitallium^®^, a new alloy of cobalt, chrome, and molybdenum (CoCrMo), was developed, and in 1938, Smith-Petersen incorporated it in his mould arthroplasty [14,18,19]. This allowed movement between the cup and the bone surfaces of the acetabulum and the femoral head and neck and since then Vitallium^®^ mould arthroplasty has been widely performed. Its survival rate and functional outcome were inferior to total hip arthroplasty, and it was found to provide long-term function of the hip joint [15,18,20], but the general material performance and implant survival turned out to be unpredictable and poor [20,21,22,23]. Although Vitallium^®^ was found to be an inert and durable material for this type of surgery, its surface characteristics were less than adequate [24].

Further efforts to explore new materials for joint arthroplasty led the Judet brothers to introduce an acrylic femoral resurfacing implant in 1946 [25]. In this technique, also called hemiarthroplasty, the artificial femoral stem was inserted into the cavity of femoral marrow with or without any kind of cementing. The Judet hip yielded favorable early results; this implant was poorly tolerated, and acrylic wear debris elicited an osteolytic reaction within the hip joint and surrounding tissue, leading to acetabular erosion and implant failure [25]. The acrylic material was soon discarded in favor of CoCrMo.

Sir John Charnley, in the 1950s, introduced hip resurfacing of the low friction material, Teflon (polytetrafluoroethylene, PTFE), to produce thin shells, which were used to “resurface” the femur and acetabulum. In this design, the acetabulum was lined with a thin shell of the material, while the head of the femur was similarly covered with a hollow sphere. It was hoped that the motion would take place preferentially between the two slippery PTFE surfaces rather than between one of the PTFE surfaces and the bone to which it was attached. Charnley assumed that if the PTFE implant remained stationary in relation to the subjacent bone, there seemed some hope that mechanical bonding might improve if bone grew into irregularities provided for this purpose [26]. This design ended shortly, causing postoperative osteolysis at a very early stage due to PTFE wear debris, leading to catastrophic failure in many cases [27].

Further developments were undertaken to improve on the early failures. In 1964, Townley, based on Judet and Judet’s design, developed the CoCrMo femoral component to articulate with a polyurethane acetabular cup [28]. Due to the ultimately demonstrated poor wear characteristics of the polyurethane cup and significant osteolysis, it was substituted with polyethylene (PE). Since it still demonstrated a high failure rate, the PE cup was replaced by a CoCrMo acetabular component. Although there are known earlier examples of the use of a metal-on-metal bearing couple applied in hip replacement, see Wiles in 1938 [29] or Haboush in 1951 [30], Müller and Boltzy were the first who published their results and are recognized as the metal-on-metal resurfacing arthroplasty pioneers [31].

Their initial concept featured a femoral component made from CoCrMo articulating with small Teflon or PE pads, known as “sliding bearings”, fixed to the acetabular cup’s inner surface. In 1968, Müller [32] shifted away from metal-on-metal articulation, opting instead for a metal-on-PE prosthesis with a curved stem. However, a high early re-operation rate of 50% prompted a revision of this approach [33]. In a similar vein, Gérard developed an HRA system in 1970, which utilized both a CoCrMo femoral head and acetabulum, allowing movement between the components as well as between the components and the surrounding bone [34]. In 1972, PE was implemented as a bearing material in the acetabular cup of this system. Nonetheless, this was discontinued in 1975 following reports about enhanced wear and osteolysis [34,35].

Also, in 1972, Nishio combined a Urist cementless acetabular cup [36] with his own femoral head of CoCrMo, which due to the growing trend for metal-on-PE designs in 1975, was substituted with the acetabular component with a PE-lined cementless socket [37,38]. In 1976, Salzer used the first ceramic-on-ceramic resurfacing endoprosthesis, which had both components made of alumina (Al_2_O_3_) [39]. Its acetabular component had three pegs for primary stability, and the femoral cup was twisted on to the prepared head. This solution was soon abandoned due to the high rates of loosening resulting from the lack of secondary fixation.

The high failure rates of these early-generation devices were the consequence of unproven material selection, the limitations in materials properties, and manufacturing of the time, crude implant design, lack of solid implant fixation at the bone–implant interface, and unrefined surgical techniques and instrumentation. These resurfacing arthroplasties had an unacceptably high wear rate resulting in failure secondary to osteolysis (i.e., aseptic loosening or inflammatory bone resorption) [40,41,42].

In the development of resurfacing implant generations of the 1970s and 1980s, particular attention has been paid to improved materials and enhanced fixation strategies to improve survivorship. New bearing materials were incorporated into hip resurfacing prostheses, and the concept of cementing components to ensure initial rigid fixation of endoprosthesis components within the bone became a way of eliminating issues associated with current cementless designs.

In 1971, Trentani [43] in Italy and Furuya [44] in Japan independently carried out the first cemented double-cup arthroplasty. Trentani and Paltrinieri developed hip resurfacing with a cemented stainless steel femoral head articulating with an acetabular ultra-high molecular weight polyethylene (UHMWPE) cup. Furuya implanted resurfacings using a stainless steel acetabular component articulating with a high-density polyethylene (HDPE) femoral component fixed with cement. In 1972, Freeman [45,46] first implanted a cemented double cup arthroplasty consisting of an HDPE femoral head coupled with a CoCrMo acetabular cup, but in 1974, the design was modified to consist of a CoCrMo femoral head and an HDPE cup. In 1973, Eicher and Capello [47] developed a cemented hip resurfacing using a metal femoral and a polyethylene acetabular component.

Similar solutions in which cemented resurfacing endoprostheses included metal or ceramic femoral heads articulating against polyethylene acetabular cups and a reversed design where a polyethylene head articulated against a metal cup were introduced independently by Wagner [48], Amstutz [2,49,50], Eicher and Capello [47], and Tanaka [51]. These designs also failed, and their poor performance and outcomes were explained by stress shielding, significant loss of blood supply, and consequential compromise to the femoral head [52,53].

In the early 1980s, the development of cementless fixation for resurfacing endoprosthesis components derived from the belief that cement was the main cause of implant failure [54].

In 1982, Amstutz introduced a plasma-sprayed (PS) metal-backed polyethylene acetabular component for use with cement, and in 1983, he implanted the first cementless resurfacing arthroplasty with a Ti-6Al-4V femoral component articulating with modular UHMWPE acetabular liners and a porous backing of pure titanium (Ti) mesh [49,50].

In 1987, Amstutz et al. presented another cementless, porous surface replacement. In this design, porous-coated or sintered beads coated with a metallic acetabular shell was used for bone fixation on both the acetabular shell with the UHMWPE insert and the CoCrMo porous-coated femoral component [55]. Although these designs achieved adequate initial cementless fixation, there were more failures with the femoral than the acetabular component due to the high wear rate of the thin polyethylene line [56,57].

In 1989, Buechel and Pappas introduced a Ti-6Al-4V modular acetabular component mated with a Ti-6Al-4V femoral head coated with a titanium nitride ceramic material. Although their laboratory simulations on the use of titanium nitride ceramic film against polyethylene in a joint couple were encouraging [58], the case report at the 11-year follow-up showed severe metallosis with catastrophic wear on the polyethylene liner [59].

A chronological review of early generations of resurfacing arthroplasty endoprostheses is presented in Table 1.

The results of hip resurfacing in the 1970s and 1980s were disappointing. Failures were the result of poor materials (conventional polyethylene was susceptible to wear [60,61]), poor implant design, inadequate instrumentation, and an imprecise surgical technique [62]. The extensive damage to the acetabulum was partially due to the significant bone removal needed for the acetabular component and its cement mantle, largely attributed to periprosthetic osteolysis. Moreover, the combination of a large articulation diameter with thin polyethylene cups or liners resulted in rapid deterioration and the generation of a considerable amount of biologically active particles, causing bone loss and loosening of the implant. Failures of hip resurfacing in the 1970s and 1980s were also attributed to avascular necrosis of the femoral head and acetabular component loosening due to high frictional torque, but with present knowledge, it is clear that the production of large volumes of biologically active particulate wear debris induced osteolysis that led to bone loss and implant loosening and caused a high incidence of fractures of the femoral neck [63]. The consistency of peri-implant bone destruction with wear particle-induced osteolysis, not avascular necrosis, was confirmed by the retrieval studies carried out by Howie et al. [64] and Campbell et al. [65]. It led to these solutions of hip resurfacing being largely abandoned by the mid-1980s [62].

## 3. Characteristics of Contemporary Hip Resurfacing Arthroplasty Endoprostheses

Resurfacing arthroplasty has been experiencing a renaissance since the early 1990s. The first two designs to appear were introduced in the early 1990s by Wagner [66] and McMinn [67]. From this time on, all further hip resurfacing devices used exclusively CoCrMo metal-on-metal bearings. Both of these first systems were cementless. Wagner’s endoprosthesis had a threaded internal geometry of the femoral component and a grit-blasted Ti surface coating at the bone interfaces, while McMinn’s endoprosthesis had anti-rotation ridges and a short epiphyseal stem to assist with femoral component alignment and stability; the first was coated with hydroxyapatite (HA) and later press fit, while the acetabular component had HA coating and peripheral fins for rotational stability. The experience gained with these solutions has shown the enduring fixation of the acetabular components [68] and improved results in terms of loosening at the early stage [69] but has demonstrated poor outcomes in longer-term follow-up [54,70,71]. The Wagner system was discontinued, while subsequent modifications to the McMinn design involved cement fixation of both components, and then the hybrid configuration evolved into the development of the Cormet™ (Corin Group, Cirencester, UK) in 1997 and current Cormet 2000 (Corin Medical Ltd., Cirencester, UK) in 2007, as well as Birmingham Hip Resurfacing (BHR)™ (Smith & Nephew, Memphis, TN, USA) endoprostheses in 1997. Meanwhile, Amstutz began a series of innovations that culminated in the Conserve Plus™ (Wright Medical Technology Inc., Arlington, TN, USA) in 1993 and began implanting it at the end of 1996 [71]. It was a hybrid (i.e., porous fixation with sintered beads on the acetabular side and cemented on the femoral side) [54].

At the turn of the 21st century, most hip resurfacing systems were hybrid with a thin-walled one-piece cementless acetabular component and a cemented femoral component. The femoral components featured a short epiphyseal stem designed for alignment during insertion.

These systems offered several benefits, such as enhanced durability in fixation, reduced wear, improved bone tissue protection, and a decreased rate of complications, particularly fractures and sprains. Numerous clinical studies and joint registry reports provided extensive evidence indicating positive outcomes and survival of surface implants. They proved to be a success, with 96%, 92%, and 88.5% survivorship at ten years for Cormet, BHR, and Converse, respectively [72,73,74,75,76].

Following these successes, in the early 2000s, numerous hybrid hip resurfacing systems emerged, e.g., the Durom™ (Zimmer Inc., Warsaw, IN, USA) introduced in 2001, the Articular Surface Replacement (ASR™) (DePuy Orthopaedics Inc., Warsaw, IN, USA), introduced in 2003, the Icon™ (IO International Orthopaedics Holding, Geisingen, Germany) and the ReCap™ (Biomet Inc., Warsaw, IN, USA), both introduced in 2004, the ADEPT™ hip resurfacing system (Finsbury Orthopaedics Ltd., Leatherhead, UK), introduced in 2005, the MITCH hip resurfacing system (Stryker, Kalamazoo, MI, USA), introduced in 2006, the ROMAX^®^ Resurfacing System (Medacta, Castel San Pietro, Switzerland), introduced in 2008, the DynaMoM hip resurfacing prosthesis (Tornier, Saint-Ismier, France), introduced in 2008, and the Minimally Invasive Hip Resurfacing (MIHR) International^®^ metal-on-metal (MoM) hip system (Comis Orthopaedics Ltd., Birmingham, UK), introduced in 2009 [77]. A list of contemporary generations of HRA endoprostheses is presented in Table 2.

Instead of all implants having a CoCrMo-on-CoCrMo bearing, the Advanced Ceramic Coated Implant Systems (ACCIS™) (Van Straten Medical, The Netherlands; Implantcast, Buxtehude, Germany), introduced in 2004, has titanium–niobium–nitride (TiNbN) ceramic surfaces engineered by physical vapor deposition (PVD) to minimize wear and prevent tribocorrosion and metal ion release. Total hip resurfacing represented the fastest-growing section in orthopedic surgery [106,107]. Although some early clinical follow-up studies of this system demonstrated promising results [108], there were also results reporting catastrophic failure of the prosthesis, and an unacceptably high revision rate was demonstrated due to unknown causes that led to cease implanting the ACCIS™ [109,110].

Another step towards biomimetics was the series of ESKA hip resurfacing systems developed by ESKA Implants GmbH & Co. (Lübeck, Germany) beginning in 2006. The first was a metal-on-metal ESKA-BIONIK^®^ hip resurfacing system (also known as Biosurf^®^ hip resurfacing), which has a unique bearing surface hydrodynamic lubrication in the bearings through the concavo–convex pattern designed to reduce abrasive wear. The pattern provided circumferentially distributed escape dimples for wear particles and improved lubrication by providing room for the lubricant [111]. The acetabular component had a spongiosa metal structured surface called Spongiosa Metal^®^ made of a CoCrMo with a titanium niobium (TiNb) coating or an HA coating, available upon request, for cementless anchorage through osseous integration that proved to have excellent long stability in clinical trials [96,97]. The cementless anchorage via the Spongiosa Metal^®^ was also applied in the case of the femoral component. Further, in 2007, based on the composite material ENDOCERAM^®^, consisting of a polyurethane matrix and a mixed-in glass ceramic powder [100,112], the ceramic-on-ceramic ESKA-CERAM^®^ hip resurfacing system was launched. The ESKA resurfacing implants were the only designs with a cementless acetabular shell in combination with a modular ceramic insert.

Research indicates that the use of cement does not consistently ensure long-term stability of endoprostheses in bones. The most common (ca. 75% of observed) complications of currently used cement resurfacing arthroplasty include the resorption of periarticular bone tissue, loosening at the bone–cement–implant junction zone, migration of endoprosthesis components, and femoral fractures [113,114,115,116]. Additionally, stress-shielding areas near the short stem of the femoral component often lead to loosening and migration [116,117,118,119,120,121]. In cemented HRA, while cement initially anchors the femoral component, it to penetrates deeply into the femoral head’s cancellous bone. Often, the area affected by cement penetration exceeds 30% of the femoral head’s total volume (some studies report over 50% [122]), leading to reduced local blood flow. This, in turn, weakens the internal microstructure of the cancellous bone in the femoral head, resulting in various complications [123,124,125,126,127,128,129].

Currently, available HRA systems exhibit a range of survival rates over five years, from a high of 97.1% to a low of 80.9% [10]. This variability has raised safety concerns regarding certain resurfacing arthroplasty endoprostheses. As a consequence, systems such as the ASR™ by DePuy Orthopaedics Inc., based in Warsaw, IN, USA, have been withdrawn from the market due to their significant rate of postoperative complications [130].

The predominant reason for early HRA failures, accounting for approximately 35% of necessary revision surgeries, is femoral neck fracture [131,132]. Additionally, aseptic loosening of either the femoral or acetabular components is another frequent reason for HRA failures [133,134]. Aseptic bone necrosis (osteonecrosis), often linked with periprosthetic fractures or identified as a contributing factor to such fractures after HRA [116], is also seen as a consequence of using cement for endoprosthesis component fixation [135] or is due to intraoperative damage to the blood vessels supplying the femoral head [136]. The heat generated during cement polymerization can severely damage the surrounding implant tissue, leading to the collapse of the femoral head [137,138]. Moreover, as previously noted, the implantation process of the femoral HRA endoprosthesis component typically forces substantial amounts of cement into the cancellous bone of the femoral head, creating a thick cement layer [139].

Aseptic bone necrosis is often seen in the early and middle stages following hip surgery, typically linked to either reduced blood flow to the femoral head or heat damage incurred during the operation [140]. Zustin et al. [140] conducted a histological analysis of 123 bone–implant samples from various resurfacing endoprostheses systems, including ASR™ by DePuy Orthopaedics, BHR™ by Smith & Nephew, Cormet™ by Corin Group, Durom™ by Zimmer Inc., and ReCap™ by Biomet Inc. These samples were collected from patients who had diagnoses other than osteonecrosis prior to surgery. The study found that osteonecrosis occurred in 88% of the cases, often linked with periprosthetic fractures. Of the revisions examined, 85 were due to periprosthetic fractures, with 60% of these fractures exhibiting complete bone necrosis near the fracture line, which are thus classified as post-necrotic fractures. Additionally, 8% of the revisions were due to the loosening of the acetabulum, and the remaining 23% were for various other reasons, including groin pain related to the femoral component. The majority of bone–implant specimens analyzed displayed extensive aseptic necrosis histologically, identified as the reason for 46% of all failures, particularly those linked to post-necrotic periprosthetic fractures and the collapse of the femoral head [141]. Steiger et al. [142] reported that, excluding infections, the leading causes for the primary revision following HRA are hip fractures (43%), loosening/lysis (32%), metal allergic reactions (7%), and pain (6%). Therefore, in cases involving serious postoperative complications, the primary revision focused on the femoral component accounts for 62% of all such revisions in the procedures mentioned [142].

ReCap™ is the only fully porous-coated femoral component currently available. The seven-year follow-up study carried out by Gross [143] suggested that cementless femoral fixation with a short epiphyseal stem may be a viable alternative to the cement fixation of HRA and pointed out that a study on a larger number of patients should be warranted.

**Figure 1:**
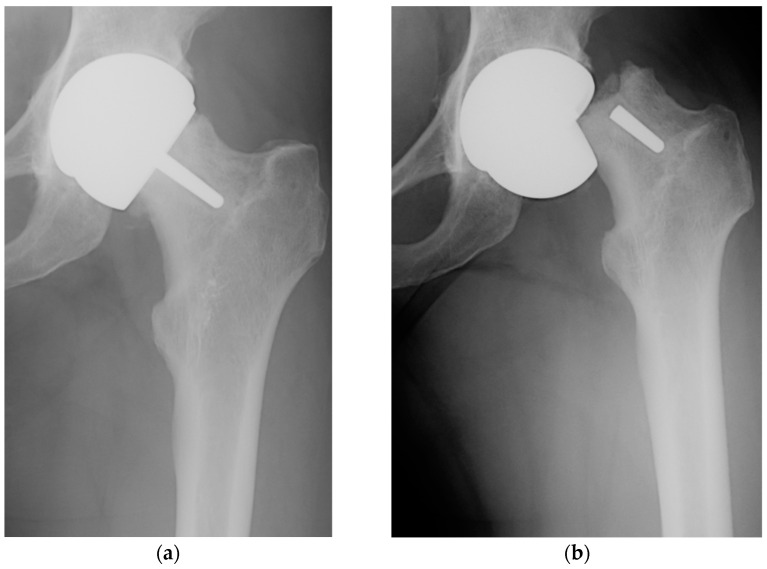
Anteroposterior radiographs demonstrating the Birmingham Hip Resurfacing system in situ (**a**) with femoral component loosening and (**b**) femoral neck fracture after one month.

In Figure 1, we present, from our own experience, the roentgenogram of a patient (female, 52 y.o.) with a perceived limp requiring a cane for ambulation, showing the loosening and fracture at the femoral neck that occurred after one month.

It should be noted that the cementless femoral fixation applied in the ReCap™ using a porous coating with sintered beads allows only shallow ingrowth of bone tissue into the pore space of the coating, and this requires the use of a short epiphyseal stem to ensure the proper fixation of the cap. Further studies [144,145] justify the validity of striving for more biomimetic bone ingrowth fixation methods in the case of the femoral component of hip resurfacing versus non-biomimetic approaches in terms of the cemented fixation methods.

## 4. First Biomimetic Resurfacing Arthroplasty Endoprosthesis with the Multi-Spiked Connecting Scaffold

A significant shift in the design philosophy for HRA endoprostheses emerged with the introduction of the original multi-spiked connecting scaffold (MSC-Scaffold) concept by Rogala [146,147,148]. This concept moved away from the traditional cemented hip endoprostheses with short epiphyseal stems towards a biomimetic, stemless, and entirely cementless design, which preserves periarticular bone tissue. The MSC-Scaffold employs a spike-palisade system that integrates the components of resurfacing arthroplasty HRA endoprostheses with the intertrabecular space of the periarticular cancellous bone. The prototype of the MSC-Scaffold was developed by a bioengineering clinical research team under the auspices of two research grants from the National Science Centre Poland (No. 4T07C05629 and No. N518412638, led by R. Uklejewski) and further research. This represents a pivotal innovation in the fixation of resurfacing endoprosthesis components within the periarticular trabecular bone. A comprehensive overview of the bioengineering research conducted on the biomimetic MSC-Scaffold prototype for a new generation of HRA endoprostheses can be found in a recent monograph [149].

**Figure 2:**
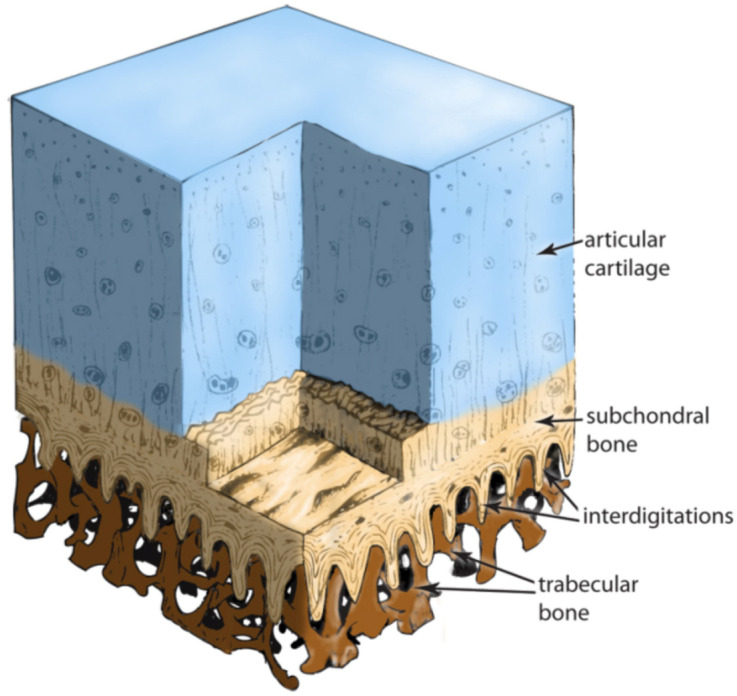
Histological scheme showing the hyaline cartilage and the subchondral bone with its interdigitations, anchoring among the trabeculae of the cancellous bone tissue [149].

The MSC-Scaffold’s spikes are designed to imitate the natural interdigitation system of the subchondral bone, which intricately weaves into the trabeculae of the surrounding cancellous bone. This design facilitates a gradual structural and biomechanical transition between the joint’s distinct morphological elements—the articular cartilage and the surrounding trabecular bone tissue of the epiphysis. Illustrated in Figure 2, the scheme shows how the subchondral bone, through its system of interdigitations, interlocks with the trabeculae of the periarticular cancellous bone. This unique interlocking mechanism forms a vital transition zone that naturally secures the articular cartilage of diarthrodial joints to the periarticular cancellous bone. Moreover, the spacing between the spikes in the MSC-Scaffold prototype is designed to allow for the growth of new bone tissue. This space acts as an osteoconductive interface, promoting the integration of periarticular trabecular bone tissue in diarthrodial human joints undergoing resurfacing arthroplasty with these endoprostheses [149].

The MSC-Scaffold facilitates the initial setting of endoprosthesis components within the bone, stimulating the bone tissue growth in its interspike spaces, leading to osseointegration. This process securely anchors the resurfacing endoprosthesis components within the periarticular bone for long-term stability. During surgery, the surgeon inserts the MSC-Scaffold spikes into the bone to a specific depth for initial mechanical stability, while the subsequent growth of new bone tissue in the spaces between the spikes provides final biological fixation during the patient’s postoperative rehabilitation. The MSC-Scaffold’s non-cemented approach to fixing HRA endoprostheses in a physiologically optimal manner could potentially reduce complications associated with bone cement use. When implanting the biomimetic MSC-Scaffold in the cancellous bone’s intertrabecular space, the spikes in a controlled fashion disrupt the bone’s trabecular microarchitecture to a beneficial osteoinductive degree. This disruption prompts adaptive remodeling and bone tissue development in the interspike areas. Furthermore, the spikes are designed to integrate with the cancellous bone’s trabecular bioconstruction, effectively dampening vibrations from dynamic joint loads and enhancing the biomechanical stability of the endoprostheses in bone tissue. This integration helps prevent the spraining and loosening of the implanted components [149].

Designed according to above-described assumptions, the MSC-Scaffold prototype facilitates the in vivo infill of its interspike spaces with newly grown trabecular bone tissue, reflecting the natural microstructure, a process not achievable with cemented HRA. This biomimetic fixation device and method for the HRA endoprosthesis components within the periarticular bone suggests that the mechanical load distribution within the periarticular bone will closely resemble that in a natural hip joint. The biomimetic MSC-Scaffold prototype aims to replicate in the implant–bone interface the transfer of mechanical loads occurring in a natural joint, which leads to almost natural biodynamics and bone tissue remodeling around the implant. Consequently, effective osteoconduction is anticipated, promoting bone tissue growth into the interspike space of the MSC-Scaffold. Additionally, the macrodimensions of the femoral component’s bearing part are designed to conserve the posterolateral and medial epiphyseal femoral arteries (*subcapsular arteriae retinaculares: superior and inferior*) of the femoral head. It ensures the preservation of physiological blood supply, meaning the preservation of key factors for adequate remodeling of the femoral head’s trabecular bone [149].

**Figure 3:**
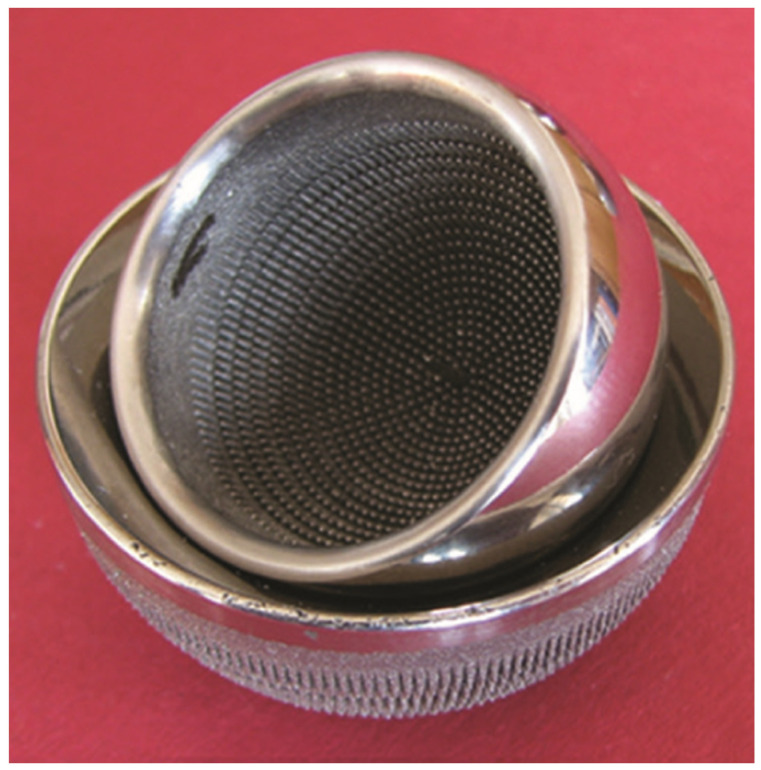
Prototype of hip resurfacing arthroplasty endoprosthesis with the multi-spiked connecting scaffold (MSC-Scaffold) [149].

Initial attempts at producing MSC-Scaffold prototypes through conventional subtractive manufacturing methods, such as die-sinking electrical discharge machining, were unsuccessful. This led to the conclusion that the fabrication of MSC-Scaffold for anchoring the components of joint resurfacing endoprostheses, as an integral part of these devices, necessitates the use of additive manufacturing technologies. Despite these methods being relatively obscure and not widely accessible in the mid-2000s, particularly for producing biocompatible metals and alloys [150], efforts were made to explore and assess the technological capabilities of fabricating the MSC-Scaffold using available additive technologies. Experiments with Selective Laser Sintering (SLS) and Electron Beam Melting (EBM) uncovered numerous unacceptable flaws in the early prototypes [151]. Further exploration to achieve manufacturing standards that ensured uniform density and mechanical properties akin to homogeneous materials led to the identification of Selective Laser Melting (SLM) as a suitable technology for producing prototype HRA endoprosthesis with the MSC-Scaffold [152,153,154]. An illustration of the HRA endoprosthesis prototype featuring the MSC-Scaffold is provided in Figure 3.

The development of the MSC-Scaffold prototype involved refining its structural and osteoconductive characteristics within the interspike space. This process led to a reevaluation of the initial design assumptions of the MSC-Scaffold, highlighting crucial insights into compensating for the revealed technological constraints of SLM technology in future design strategies. This meant focusing on enhancing pro-osteoconductive structural functionality for the effective design of subsequent MSC-Scaffold prototypes [155]. Biological verification of the functionalized MSC-Scaffold with human osteoblasts demonstrated that its interspike region offers an efficient space for the proliferation and spread of osteoblasts. These cells exhibited a propensity to form a three-dimensional intercellular network, reflecting the characteristic biostructure of lamellar bone tissue found in the trabeculae of trabecular bone [156].

Early pilot implantations of structurally functionalized MSC-Scaffold preprototypes in an animal model (swine; breed: Polish Large White) demonstrated no postoperative issues, such as implant loosening, migration, or other early complications. Histopathological analysis revealed that the majority of the interspike spaces were filled with newly formed and mineralized bone tissue. It secured the primary biological fixation of the MSC-Scaffold preprototypes in the periarticular trabecular bone [157]. These findings also highlighted the necessity for improved bone contact, suggesting the surface of the MSC-Scaffold spikes be coated with calcium phosphate (CaP) to more closely mimic the biochemical properties of native bone biomineral (HA).

Research into electrochemical cathodic deposition of CaPs on the spike surfaces initially used constant current densities [158] and then moved to a potentiostatic electrodeposition process, followed by immersion in simulated body fluid [159]. This led to the development of efficient process parameters that ensured biomineral coverage of the bone-contacting surface, with a Ca/P molar ratio similar to that of native bone HA. Testing the CaP-coated surfaces with human osteoblasts indicated that such modifications are favorable for bone cell proliferation, alkaline phosphatase activity, and hence, the mineralization and osteoinduction/osteointegration potential of the MSC-Scaffold. The distance between the MSC-Scaffold spikes was found to be a significant factor affecting alkaline phosphatase activity. Biointegration assessments of these prototypes implanted into the knee joints of swine (breed: Polish Large White), performed eight weeks post-implantation, showed a scaffolding effect with the majority of interspike spaces filled by newly formed and remodeled bone tissue, ensuring primary biological fixation. Notably, based on calculations performed on micro-CT reconstructions of explanted knee joints, a higher percentage (about 12%) of trabeculae was observed between the spikes of the CaP-modified MSC-Scaffold [159].

Biomechanical studies on the bone–implant system relevant to the MSC-Scaffold’s design were conducted, with numerical simulations identifying key geometric features that ensure physiological load transfer from the MSC-Scaffold to the surrounding bone [160]. A validated numerical model, developed and tested with micro-CT-assisted mechanical tests, simulated the MSC-Scaffold embedded in cancellous bone material [161]. Analysis of the Huber–Mises–Hencky (HMH)-reduced stress distribution from these simulations confirmed that the structural biomimicry of the MSC-Scaffold prototype enables a physiologically uniform surface transfer of the mechanical load from the spikes to the trabeculae of the periarticular trabecular bone. In conclusion, the early postoperative biomechanical load capacity (loadability) of the articular surface of the cementless and stemless HRA endoprosthesis with the MSC-Scaffold is considered to be *the crucial design criterion* for such endoprostheses. In the early postoperative rehabilitation period, the controlled loading by the patient of the joint with such endoprosthesis will enable bone tissue to grow in the MSC-Scaffold interspike spaces and will ensure the final bone–implant fixation.

Pilot studies on the MSC-Scaffold in an animal model involved the implantation of prototype partial knee resurfacing endoprostheses with the MSC-Scaffold to ten experimental animals (swine; breed: Polish Large White) [162]. Micro-CT analyses of specimens explanted from these animals eight weeks after surgery revealed (1) the interspike space occupied by newly formed and remodeled mature bone tissue, (2) initial stages of osseointegration in the form of bone tissue creeping substitution identified in deeper areas of the MSC-Scaffold and (3) the distribution of different radiological phases changing as a function of the distance from the bases of the spikes [162]. These results demonstrate that the biomimetic fixation method via the MSC-Scaffold ensures a cementless and stemless anchoring of the endoprosthesis component in the trabecular bone.

With the completion of preclinical bioengineering research on the MSC-Scaffold, the next phase of clinical surgical research in humans is set to begin, involving experimental surgical treatments of damaged knee and hip joints using prototype resurfacing endoprostheses with the biomimetic MSC-Scaffold.

## 5. Summary and Final Remarks

The goals of HRA are restoring joint anatomy, biomechanics, and function while prolonging the life of a patient with endoprosthesis by preserving bone stock for easy further possible revision arthroplasty in the form of traditional total hip arthroplasty. Over decades of its history, the designs of HRA endoprostheses have undergone a variety of technological innovations in terms of evolution in materials and fixation techniques, which often resulted in promising outcomes and the extension of implant survival. Although these early hip resurfacing designs showed good early functional results, they most disappointingly failed in longer follow-ups due to the inadequacy of materials, poor implant design, inadequate instrumentation, and an imprecise surgical technique. Most studies on the failures of contemporary resurfacing arthroplasties, in which practically all femoral components are fixed with cement, lead to the conclusion that femoral cement failure is the most common late cause of failure in hip resurfacing. In osteonecrotic femoral heads, there is a lack of adequate blood supply, which prevents peri-implant bone regeneration and deepens the deterioration of bone surrounding the implant leading to dysfunction in terms of load transfer in the artificial joint. This often led to femoral loosening, migration, or fractures that resulted in ceasing some of the contemporary designs of resurfacing endoprostheses. Since cement was a confirmed causative factor in femoral failures after resurfacing, the need arises for biomimetic bone–implant fixation methods that would provide a biomimetic structure and biomechanics of the artificial joint.

The newly developed fixation method for resurfacing arthroplasty endoprostheses is distinguished by the biomimetics inherent in its MSC-Scaffold, which are characterized by the following:

(1) Resemblance to the microstructure of the periarticular subchondral and cancellous bone tissue;
(2) Conservation of the femoral head’s posterolateral and medial epiphyseal arteries (*subcapsular arteriae retinaculares: superior and inferior*);
(3) Facilitation of a load transfer that mimics natural bone biomechanics, reflecting the mechanical behavior observed in a natural hip joint where the load is transmitted through the trabeculae in the femoral head and neck, continuing along the femoral shaft.

Against many observed failures of the standard fixation technique of contemporary HRA endoprostheses, where the femoral component is fixed with the use of cement, our prototype of a biomimetic MSC-Scaffold can be regarded as a promising breakthrough in bone–implant advanced interfacing in joint resurfacing arthroplasty endoprostheses fixation techniques. The MSC-Scaffold prototype manufactured with modern advanced laser additive technology opens a new generation for the first biomimetic resurfacing joint endoprostheses.

This new design type of completely cementless and stemless resurfacing arthroplasty endoprostheses of the hip joint (and other joints), where endoprosthesis components are embedded in the surrounding bone via the prototype biomimetic multi-spiked connecting scaffold (MSC-Scaffold), initiates the first of all generations of biomimetic endoprostheses of diarthrodial joints.

## Figures and Tables

**Table 1:** Early resurfacing arthroplasty endoprostheses (chronological review).

Surgeon/Designer	Introduced	Materials	Fixation	Reference
Smith-Petersen	1923	Glass		[14]
Smith-Petersen	1925	Viscaloid		
Groves	1927	Ivory		[16]
Smith-Petersen	1933	Pyrex		
Smith-Petersen	1937	Bakelite		
Smith-Petersen	1938	Vitallium^®^ (CoCrMo)		[14,18,19]
Judet and Judet	1946	Acrylic		[25]
Charnley	1951	Teflon		[26,27]
Townley	1964	CoCrMo femoral head/polyurethane acetabular cup CoCrMo femoral head/PE acetabular cup		[28]
Müller and Boltzy	1968	CoCrMo femoral head/CoCrMo acetabular cup		[31]
Gérard	1970	CoCrMo femoral head/CoCrMo acetabular cup CoCrMo femoral head/PE acetabular cup		[34,35]
Trentani & Paltrinieri	1971	stainless steel femoral head/UHMWPE acetabular cup	Cemented	[42]
Furuya	1971	HDPE femoral head/stainless steel acetabular cup	Cemented	[43]
Nishio	1972	CoCrMo femoral head/CoCrMo acetabular cup	Cementless	[37]
Nishio	1975	CoCrMo femoral head/polyethylene acetabular cup	Cementless	[38]
Freeman	1972 1974	HDPE femoral head/CoCrMo acetabular cup CoCrMo femoral head/UHMWPE acetabular cup	Cemented Cemented	[45,46]
Eicher and Capello	1973	CoCrMo femoral head/UHMWPE acetabular cup	Cemented	[47]
Wagner	1974	CoCrMo femoral head/UHMWPE acetabular cup Al_2_O_3_ femoral head/UHMWPE acetabular cup	Cemented	[48]
Amstutz	1975	CoCrMo femoral head/UHMWPE acetabular cup	Cemented	[49,50]
Salzer	1976	Al_2_O_3_ femoral head/Al_2_O_3_ acetabular cup	Cementless	[39]
Tanaka	1978	CoCrMo femoral head/UHMWPE acetabular cup	Cemented	[50]
Amstutz	1982	CoCrMo femoral head/UHMWPE PS metal-backed acetabular cup	Cementless (Press fit)	[49,50]
Amstutz	1983	Ti-6Al-4V femoral head with sintered titanium fiber mesh/UHMWPE liner with porous backing of pure titanium mesh	Cementless	[49,50]
Amstutz	1987	Porous-coated CoCrMo femoral head UHMWPE insert Porous-coated or sintered bead-coated metallic acetabular cup	Cementless	[55]
Buechel and Pappas	1989	Titanium nitride-coated Ti-6Al-4V femoral head, UHMWPE liner, Ti-6Al-4V acetabular cup	Cementless	[57,59]

**Table 2:** Contemporary HRA endoprostheses.

System	Introduced	Femoral Component Material and Fixation	Acetabular Component Bearing Material/Bone-Contacting Material	References
Wagner’s	1991	CoCrMo cementless, press-fit	CoCrMo/grit-blasted Ti coating, cementless	[66]
McMinn’s	1992	CoCrMo cementless, initially HA coating, then cemented	CoCrMo/HA coating, cementless	[67]
Conserve Plus™ (Wright Medical Technology Inc., Arlington, TN, USA)	1996	CoCrMo cemented	CoCrMo/CrCrMo beads + HA coating, cementless	[78,79,80]
BHR™ (Smith & Nephew, Memphis, TN, USA)	1997	CoCrMo cemented	CoCrMo/CrCrMo beads + HA coating, cementless	[81,82]
Cormet™ (Corin Group, Cirencester, UK)	1997	CoCrMo cemented (cementless option with PS Ti, HA coating)	CoCrMo/PS Ti + HA coating, cementless	[83,84]
Durom™ (Zimmer Inc., Warsaw, IN, USA)	2001	CoCrMo cemented	CoCrMo/PS Ti, cementless	[85,86]
ASR™ (DePuy Orthopaedics Inc., Warsaw, IN, USA)	2003	CoCrMo cemented	CoCrMo/CrCrMo beads + HA coating, cementless	[87,88]
Icon™ (IO International Orthopaedics Holding, Geisingen, Germany)	2004	CoCrMo cemented	CoCrMo/CrCrMo beads + HA coating	[89,90]
ReCap™ (Biomet Inc., Warsaw, IN, USA)	2004	CoCrMo cemented (cementless option PS Ti-6Al-4V)	CoCrMo/PS Ti-6Al-4V + HA coating	[91]
ACCIS™ (Van Straten Medical, The Netherlands; Implantcast, Buxtehude, Germany)	2004	TiNbN-coated CoCrMo cemented fixation, cementless from 2009	TiNbN-coated CoCrMo/ PS Ti	[92]
ADEPT^®^ (Finsbury Orthopaedics Ltd., Leatherhead, UK)	2005	CoCrMo cemented	CoCrMo/CrCrMo beads + HA coating	[93,94]
MITCH (Stryker, Kalamazoo, MI, USA)	2006	CoCrMo cemented	CoCrMo/PS Ti + HA coating	[95]
ESKA-BIONIK^®^ (ESKA Implants GmbH & Co., Lübeck, Germany)	2006	CoCrMo; Spongiosa Metal^®^ (cemented option)	CoCrMo + CoCrMo insert/Spongiosa Metal^®^	[96,97,98]
ESKA-CERAM^®^ (ESKA Implants GmbH & Co., Lübeck, Germany)	2007	CoCrMo; Spongiosa Metal^®^ (CoCrMo with TiNb coating)	polyurethane/Al_2_O_3_ + polyurethane/Al_2_O_3_ insert/ Spongiosa Metal^®^ (CoCrMo with TiNb coating)	[98,99,100]
Cormet 2000 (Corin Medical Ltd., Cirencester, UK)	2007	CoCrMo cemented	CoCrMo/PS Ti + HA coating	[84,101]
ROMAX^®^ (Medacta, Castel San Pietro, Switzerland)	2008	CoCrMo cemented	CoCrMo/PS Ti + HA coating	[102,103]
DynaMoM (Tornier, Saint-Ismier, France)	2008	CoCrMo cemented	CoCrMo/PS Ti + HA coating	[104]
MIHR International^®^ (Comis Orthopaedics Ltd., Birmingham, UK)	2009	CoCrMo cemented	CoCrMo/HA coating, cementless	[105]

## Data Availability

No new data were created or analyzed in this study. Data sharing is not applicable to this article.
